# Complete regression of advanced prostate cancer for ten years: A case report and review of the literature

**DOI:** 10.3892/ol.2013.1377

**Published:** 2013-06-06

**Authors:** BING YAN, XIANZE MENG, XIAOWEI WANG, PINKANG WEI, ZHIFENG QIN

**Affiliations:** Department of Traditional Chinese Medicine, Shanghai Changzheng Hospital, Second Military Medical University, Shanghai 200003, P.R. China

**Keywords:** complete regression, erythrocyte sedimentation rate, prostate specific antigen, traditional Chinese herbal medicine, prostate cancer

## Abstract

Long-term complete regression of prostate cancer (PCa) is a rare phenomenon. The current report presents the case of an advanced PCa patient with rare clinical features. Following the generation of a definitive diagnosis, the patient was administered with flutamide treatment (0.25 g flutamide) 3 times a day, for 5 consecutive years, prior to surgical castration. Following surgery, 3.75 mg enantone was injected (i.h.) once per month for 3 months, without suspending the flutamide treatment. In addition, traditional Chinese herbal medicine was administrated immediately following surgery. Strontium-89 radiotherapy was performed for multiple bone metastases, and the multiple metastatic lesions (lung and bone) of the individual disappeared in <7 months. The patient has currently survived for >10 years with no development of castration resistance or signs of recurrence. Nadir prostate-specific antigen (PSA) levels had remained at <0.1 ng/ml following the initial treatment, and the erythrocyte sedimentation rate (ESR) value was high and had been observed to fluctuate during the treatment. The present case report considers the role of the androgen-receptor in PCa and indicates that careful interpretation of nadir PSA and ESR levels may aid in the prediction of patient prognosis.

## Introduction

Prostate cancer (PCa) is the second leading cause of mortality in the western world, but the single most common non-cutaneous malignancy in the United States, with ∼241,740 and ∼28,170, morbidities and mortalities, respectively, in 2012 (1,2). Despite an increase in available reagents for PCa treatment (3–5), the prognosis for advanced-stage patients remains discouraging, with a median life expectancy of ∼2.5 years (6). Long-term complete regression of PCa is uncommon and the complex mechanisms involved in advanced PCa are not yet understood. The current report presents the case of a patient with stage IV PCa, with rare clinical features, indicating a role for the androgen-receptor in PCa. Written informed consent was obtained from the patient.

## Case report

### Patient presentation and diagnosis

**Figure 1.**
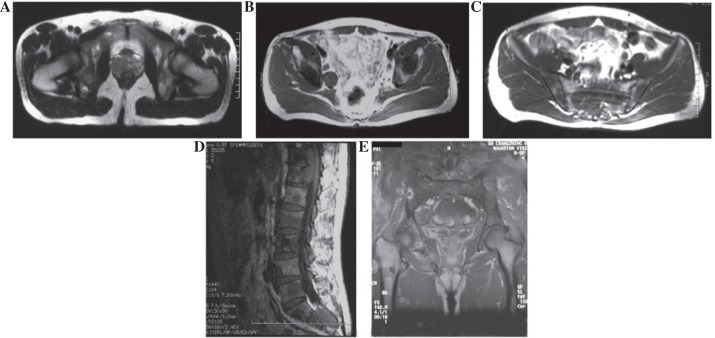
MRI of pelvis and lumbar spine (T1 weight). (A) Slightly increased prostate volume, with a less smooth fringe and non-uniform signal at the bottom of the peripheral ribbon. (B) Soft tissue nodules in the right groin. (C) Bilateral ilium showing a low signal, notably on the right. (D and E) T11–12 intraspinal tumor and bilateral thigh bone detecting low signals.

**Figure 2.**
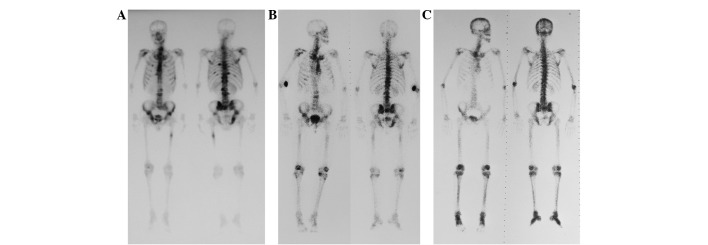
Patient bone scans. (A) Multiple skeletal metastases apparent in ribs, lumbar spine and bilateral ilium (November, 2002). (B) Evident shrinking of bone metastasis with the exception of the lumbar spine (26 March 2003) (C) Complete remission of bone metastasis (June 24, 2003).

**Figure 3.**
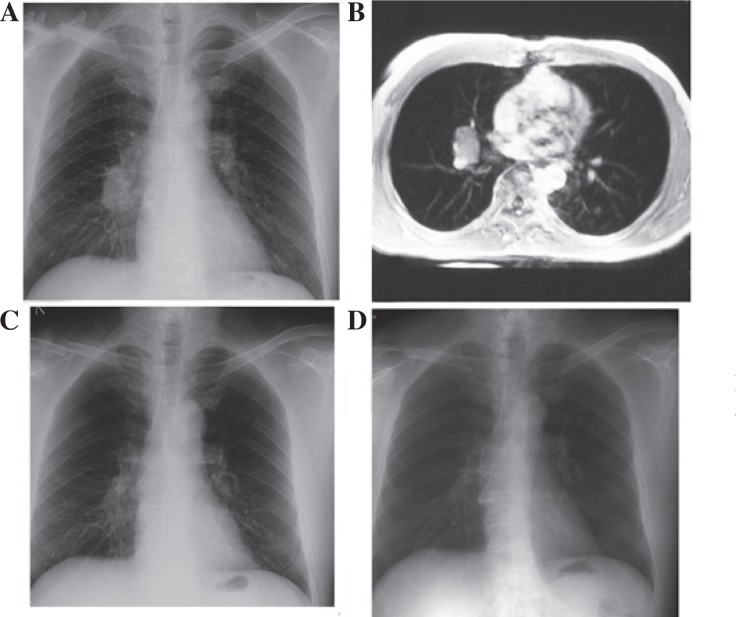
Chest radiograph and MRI of the lungs. (A) Chest radiograph identifiying a lesion in the right hilus pulmonis with a clear edge (November 26, 2002). (B) MRI identifying a 3×2-cm lobulated node in the right hilum (November 26, 2002). (C) Chest radiograph detecting no mass, but indicating the presence of a chaotic blood vessel system (December 29, 2002). (D) Normal chest radiograph (December 20, 2012).

**Figure 4.**
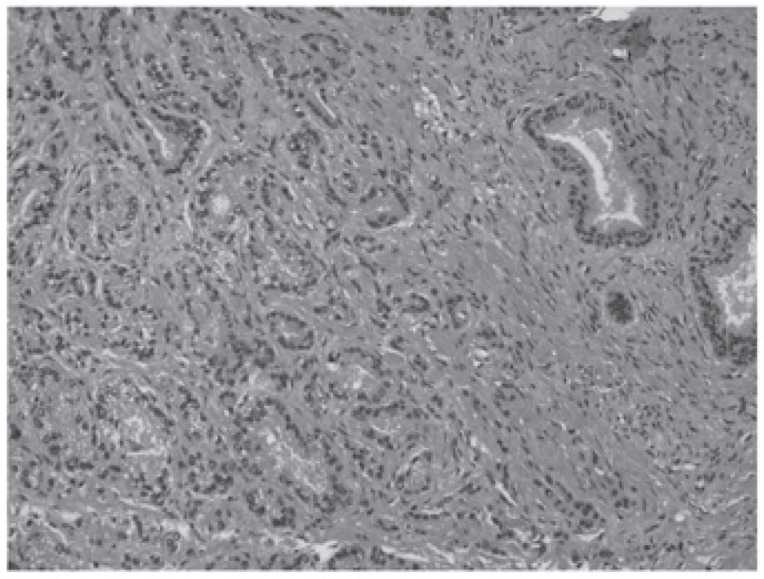
Pathology results for the prostate. Rounded cells with enlarged nuclei and an irregular gland shape, deeply stained and infiltrating the normal tissue.

A 51-year-old male with progressive weakness, dull shoulder and back pain and low-grade fevers in the afternoon (range, 37.7–38.3°C) was referred to Shanghai Changzheng Hospital (Shanghai, China) in November, 2002. Two months previously, the individual detected a mass in the right groin, which was pliable in texture with no pain upon the addition of pressure. A physical examination revealed a 2×2-cm mass in the right groin. Blood pressure, pulse and body temperature values were all within the normal range. ECG results were normal, as were results from blood and fecal tests. Prostate-specific antigen (PSA) tumor marker levels were >500 ng/ml (reference value, 0–35 ng/ml), however, other tumor markers, including α-fetoprotein (AFP), carcinoembryonic antigen (CEA), carbohydrate antigen (CA)19-9, CA12-5 and neuron-specific enolase (NSE) remained within the normal ranges. The erythrocyte sedimentation rate (ESR) was 45 mm/h and the anti-streptolysin ‘O’ and anti-rheumatoid factor test results were negative. MRI of the pelvis and the lumbar spine detected an enlarged prostate with non-uniform signals at the bottom of the peripheral ribbon, multiple infiltrating lesions in the lumbar, sacrum, pelvis and bilateral thighbone, a T11–12 intra-spinal tumor and soft tissue nodules in the right groin (Fig. 1). A bone scan revealed multiple skeletal metastases (Fig. 2A) and a chest radiograph and lung MRI identified a 3×2-cm lobulated node in the right hilum (Fig. 3A and B). A review of the patient’s medical history showed the individual had suffered from lumbar disk disease (T5-S1) for 8 years, in addition to a long-term history of smoking and alcohol use. The patient was diagnosed with advanced prostatic cancer (IV, cT4N2M1c) following an ultrasonographic-guided biopsy performed in November, 2002. Pathology results identified rounded cells with enlarged nuclei and an irregular gland shape, which were deeply stained and infiltrated the normal tissue (Fig. 4).

### Treatment and clinical course

**Figure 5.**
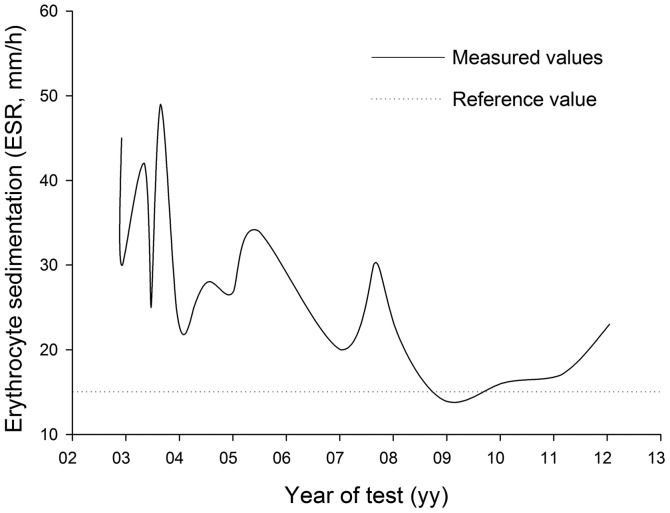
Fluctuation of erythrocyte sedimentation rate (ESR). High values were detected between 2002 and 2012, with the exception of between 2008 and 2010.

Flutamide (0.25 g) was administered (p.o.) 3 times a day prior to surgical castration in December 2002. In addition, 3.75 mg enantone was injected (i.h.) once every month, for 3 months, without suspending the flutamide treatment. A traditional Chinese herbal medicine (TCHM) was administrated immediately following surgery and at follow-up appointments (Table I). In January 2003, strontium-89 radiotherapy for multiple bone metastases was performed. Laboratory tests at that time indicated a significant decrease in PSA levels to 0.32 ng/ml, which had reached 0.03 ng/ml at the end of the month. In addition, a chest radiograph identified that the lung lesion had gone (Fig. 3C). In March 2003, a repeat chest radiograph, which detected no abnormalities, was performed and a bone scan demonstrated a marked reduction of bone metastasis (Fig. 2B). Upon first admission, the patient exhibited levels of ESR that fluctuated above normal (Fig. 5), while the PSA levels remained at <0.1 ng/ml. In June 2003, an additional bone scan revealed complete remission of the bone metastasis (Fig. 2C). Annual bone scans continued to confirm this result until the scans were stopped in June 2005.

The administration of flutamide was withdrawn in May 2007, but the use of TCHM was continued; no adverse effects were identified by the individual, with the exception of controllable hot flushes. However, no recurrence was detected at the annual follow-up appointments. During treatment, blood, urine, stool, electrolyte, biochemistry, tumor marker and hemagglutinin tests were performed and demonstrated to be within the normal ranges. When CT or MRI scans were not performed at the patient’s follow up appointments, a visceral ultrasound examination, including an examination of the prostate, was arranged and no abnormalities were detected. The patient’s most recent appointment was in December 2012, where a physical examination and a chest radiograph detected no abnormalities (Fig. 3D). An examination of the liver, gall bladder, pancreas, spleen, kidney, prostate, bladder and the lymph nodes of the bilateral groin was performed by abdominal ultrasound and were all identified to be normal. PSA and ESR levels were 0.06 ng/ml and 25 mm/h, respectively.

## Discussion

According to the international system for staging PCa, the present case was classified as clinical stage IV (cT4N1M1c) PCa. Few studies of the regression of PCa metastasis have been published and the majority describe single lesions with no records of long-term follow-up (Table II) (7–13). Therefore, in this regard, the present case is unique.

The cellular and molecular events underlying the development of PCa are not yet understood, but it has been demonstrated that the role that androgens play is significant and, as a result, anti-androgen therapy is the preferred treatment. For previously untreated and advanced PCa, anti-androgen monotherapies, including flutamide therapy, has been reported to be effective (14,15). However, only single androgen deprivation therapy (ADT) is recommended by the National Comprehensive Cancer Network (2011) for M1 patients, based on the evidence that combined- or triple-androgen blockage represents no survival benefit over castration alone (16). In the present case report, the treatment regimens conflicted with the treatment guidelines and recommendations for PCa, and the reason for the final notable results remains currently unclear. The majority of advanced PCa cases are initially sensitive to ADT, however, the magnitude of castration-induced primary regression does not predict clinical outcome (17) and patients generally develop castration resistance within a median time of 12–18 months (18). Treatment of castration-resistant PCa (CRPC) is challenging since growth of the cancer at this stage is hypothesized to be regulated by androgens, and mutations of the androgen-receptor (AR) genes are common (19,20). However, previous studies have indicated that the AR remains a significant target in patients with CRPC (21). Although results of the current case report are unclear, based on the management and clinical presentation, it may be hypothesized that androgens play a significant role in PCa.

Advances in molecular biomarkers have developed prognostic factors, allowing for improved identification of patients likely to benefit from a specific reagent and are therefore essential for selecting treatments. PSA is a well-established marker for monitoring treatment response and disease recurrence (22,23). Various parameters of PSA have been studied (24,25) for example, a nadir PSA of <4 ng/ml within 6–7 months following initial treatment has been identified to be a significant predictor of the progression time to CRPC and overall survival (24,26). Previous studies have demonstrated that 40–50 mm/h ESR at diagnosis is a marker for low-risk cancer-specific mortality (27). The present patient had a nadir PSA of 0.1 ng/ml at 2 months after the treatment, which remained low at the follow-up appointments. In addition, ESR was 45 mm/h at diagnosis. We hypothesize that these features indicate an improved prognosis. Other prognostic factors, including circulating tumor cells, have also been demonstrated as useful for predicting survival benefit following treatment for metastatic CRPC and hormone-sensitive PCa (28,29), however, results have yet to be confirmed and validated by future studies (30).

Alternative medicine is popular among cancer patients and previous studies have demonstrated that 8.4–26.5% of PCa patients use herbal remedies (31,32). TCHMs, including Realgar-Indigo naturalis and PHY906, are some of the most popular remedies and have been scientifically proven to be effective for cancer management (33–35). Results indicating that TCHMs may lead to the complete regression of cancer have been obtained in lung cancer and hepatocellular carcinoma (36,37). In the present case report, a TCHM was taken at the onset of treatment and then consistently for 4 years. Although it is hypothesized that the withdrawal of flutamide may induce a reduction of PSA in 40% of PCa patients (38), no rebound of PSA or recurrence was identified, therefore, TCHM may have a certain treatment value. The efficacy of TCHM cannot be defined in patients based on the current case report and no conclusive evidence has been obtained from randomized trials. However, the current study and others have indicated that TCHM may be an effective option for the future management of PCa.

Overall, the present case report demonstrates a role for the androgen-receptor in PCa and indicates that the careful interpretation of nadir PSA and ESR may effectively predict patient prognosis in the future.

## Figures and Tables

**Table I.** Ingredients of the traditional Chinese herbal medicine.

Chinese name	Latin name	%
Huangqi	*Astragali Radix*	13.51
Taizishen	*Pseudostellariae Radix*	13.51
Nvzhenzi	*Ligustri Lucidi Fructus*	13.51
Gouqi	*Lycii Fructus*	6.75
Biejia	*Trionycis Carapax*	4.50
QuanXie	*Scorpio*	2.70
Wugong	*Scolopendra*	4.50
Tianlong	*Gekko Chinensis*	6.75
Dilong	*Pheretima*	6.75
Banbianlian	*Lobeliae Chinensis Herba*	6.75
Banzhilian	*Scutellariae Barbatae Herba*	6.75
Jineijin	*Galli Gigerii Endothelium Corneum*	6.75
Dazao	*Jujubae Fructus*	4.50
Gancao	*Glycyrrhizae Radix Et Rhizoma*	2.70

**Table II.** Results of published case reports of the regression of metastasis in prostate cancer.

First author/s (ref)	No. of cases	Location of metastasis	Evidence of regression	Management	Follow-up record
Peyrí Rey (7)	1	Bone	Bone scan	ADT	NA
Kumar *et al* (8)	1	Eye	Not available	Hormonal therapy	NA
Hoshi *et al* (9)	1	Bone	Bone scan	Cisplatin, UFT, dexamethasone, diethylstilbestrol diphosphate	NA
Weiss *et al* (10)	1	Bone	Scintigraphy	Surgery/153Sm-EDTMP	NA
Ameur *et al* (11)	1	Brain	NA	NA	Recurrence
Gayet and Curtillet (12)	NA	Lung	NA	NA	NA
Turner and Chaudhary (13)	1	Bone	PSA/Imaging	Alternative therapies	NA

ADT, androgen deprivation therapy; 153Sm-EDTMP, Samarium-153-ethylene diamine tetramethylene phosphonate; UFT, tegafur-uracil; PSA, prostate-specific antigen; NA, not available.
